# Enhancement of Facilitation Training for Aphasia by Transcranial Direct Current Stimulation

**DOI:** 10.3389/fnhum.2020.573459

**Published:** 2020-09-11

**Authors:** Aya S. Ihara, Akiko Miyazaki, Yukihiro Izawa, Misaki Takayama, Kozo Hanayama, Jun Tanemura

**Affiliations:** ^1^Center for Information and Neural Networks, National Institute of Information and Communications Technology and Osaka University, Kobe, Japan; ^2^Rehabilitation Center, Kawasaki Medical School Hospital, Kurashiki, Japan; ^3^Department of Childhood Education, Faculty of Education, Fukuyama City University, Fukuyama, Japan; ^4^Department of Rehabilitation, Okayama Rehabilitation Hospital, Okayama, Japan; ^5^Department of Rehabilitation Medicine, Kawasaki Medical School, Kurashiki, Japan; ^6^Faculty of Rehabilitation, Kawasaki University of Medical Welfare, Kurashiki, Japan

**Keywords:** aphasia, transcranial direct current stimulation (tDCS), naming, speech, language training, neurorehabilitation, deblocking, Broca’s area

## Abstract

We aimed to enhance the performance of naming and sentence production in chronic post-stroke aphasia by tablet-based language training combined with transcranial direct current stimulation (tDCS) conducted on non-consecutive days. We applied a deblocking method involved in stimulation–facilitation therapy to six participants with chronic aphasia who performed naming and sentence production tasks for impaired modalities, immediately after a spoken-word picture-matching task for an intact modality. The participants took part in two conditional sessions: a tDCS condition in which they performed a spoken word-picture matching task while we delivered an anodal tDCS over the left inferior frontal cortex; and a sham condition in which sham stimulation was delivered. We hypothesized that, compared with the sham stimulation, the application of anodal tDCS over the left inferior frontal cortex during the performance of tasks requiring access to semantic representations would enhance the deblocking effect, thereby improving the performances for subsequent naming and sentence production. Our results showed greater improvements 2 weeks after training with tDCS than those after training with sham stimulation. The accuracy rate of naming was significantly higher in the tDCS condition than in the sham condition, regardless of whether the words were trained or not. Also, we found a significant improvement in the production of related words and sentences for the untrained words in the tDCS condition, compared with that found pre-training, while in the sham condition we found no significant improvement compared with that found pre-training. These results support our hypothesis and suggest the effectiveness of the use of tDCS during language training on non-consecutive days.

## Introduction

Aphasia is an impairment in language function resulting from brain damage mainly by left-hemispheric stroke. While speech-language therapy plays a central role in improving the function of patients with aphasia, the improvement is mild at the chronic stage. The use of transcranial direct current stimulation (tDCS), a neuromodulation technique, has been proposed to be effective in improving function in chronic aphasia (see review Shah et al., 2013; Flöel, 2014). Improvement under the application of tDCS has been reported in picture naming (Monti et al., 2008; Baker et al., 2010; Fiori et al., 2011; Flöel et al., 2011; Kang et al., 2011; Campana et al., 2015), verb naming (Fiori et al., 2013; Campana et al., 2015), repetition (Marangolo et al., 2011), verbal fluency (Vines et al., 2011), and sentence production (Marangolo et al., 2013a,b). Importantly, the improvement effect could be long-lasting for a few months after applying tDCS. Although the guidelines on the therapeutic use of tDCS published in 2017 (Lefaucheur et al., 2017) demonstrated the absence of sufficient evidence for the effect on chronic stage post-stroke aphasia, Stahl et al. (2019) recently proposed a Phase III protocol of intensive speech–language therapy combined with tDCS. Thus, the future clinical use of tDCS for aphasia is anticipated.

Many tDCS studies for chronic stroke aphasia have shown long-term improvement through the application of tDCS on multiple consecutive days (Baker et al., 2010; Fiori et al., 2011, 2013; Flöel et al., 2011; Fridriksson, 2011; Jung et al., 2011; Marangolo et al., 2011, 2013a). Although a single session of tDCS application has shown a facilitation effect in picture naming immediately after training (Monti et al., 2008; Lee et al., 2013)—but no effect in another study (Santos et al., 2017)—the long-term effect remains unclear. The results of tDCS studies on nonlinguistic functions of stroke patients suggest that the effect of single-session tDCS is short-lived; multiple sessions are probably required to bring tDCS effects to a clinically meaningful level (see review Lefaucheur et al., 2017). For aphasic patients, tDCS application on multiple consecutive days appears to be the best choice to produce long-term effects (see review Monti et al., 2013). On the other hand, to our knowledge, no studies have examined whether speech-language training combined with tDCS on multiple non-consecutive days produces a long-term effect. Most post-stroke aphasia patients have hemiplegia, and thus require the cooperation of family members for hospital visits. Intensive tDCS therapy conducted on multiple consecutive days would, therefore, place a heavy burden on aphasic patients and their families. In Japan, due to the insurance system, there is a limit to the number of medical rehabilitations that can be undertaken during the chronic stage; added to this, there are not enough hospitals and facilities where chronic aphasia patients can receive speech-language therapy rather than physical rehabilitation. We, therefore, aim in the present study to clarify the potential of speech-language training combined with tDCS for chronic stroke aphasia on multiple non-consecutive days to produce some long-lasting improvement in naming and sentence production. The provision of long-lasting effects would expand the possibility of aphasic rehabilitation using tDCS.

Here, we applied a technique for the facilitation of aphasic performance called the deblocking method (Weigl, 1981), a widely used stimulation–facilitation therapy. Deblocking supposedly reactivates the damaged language processing circuit. In cases of aphasia, the capacity for linguistic information processing varies depending on language modalities—such as comprehension, naming, repetition, oral reading, and writing. In the deblocking phenomenon, within under 10 min after responding to certain words or sentences with an intact modality (deblocking task), a patient can respond correctly in a modality which has previously been impaired (deblocked task), and the facilitation of the performance of the deblocked task lasts for over 2 days. Howard et al. (1985) demonstrated that techniques requiring patients to access the semantic representations corresponding to the target object—such as word-to-picture matching and semantic judgment—facilitated picture naming in aphasia, and the facilitation effect lasted for at least 24 h, while techniques requiring access to phonological representations—such as repetition and rhyme judgment—showed no such effect. Based on these findings, we considered that the long-lasting facilitation effect by the deblocking method would be enhanced if semantic access were to be facilitated by tDCS during the deblocking task.

Our previous study demonstrated that in healthy participants, performance in semantic retrieval can be facilitated by anodal tDCS over the left inferior frontal cortex (Ihara et al., 2015), an area proposed to be involved in controlled semantic retrieval and selection (Thompson-Schill et al., 1999; Cardillo et al., 2004; Badre et al., 2005; Bunge et al., 2005; Gold et al., 2006; Ihara et al., 2007; Grindrod et al., 2008). Marangolo et al. (2013a) demonstrated that after conversational training with anodal tDCS over the Broca’s area, compared with that with tDCS over the Wernicke’s area and sham stimulation, participants with chronic non-fluent aphasia produced more content units, verbs, and sentences. They considered the result to be attributable to the stimulation of Broca’s area, which facilitated the process of semantic retrieval and selection. In the present study, we targeted participants with chronic aphasia in whom comprehension of the spoken word was intact but naming and sentence production were impaired and designed training using the deblocking method in which they performed naming and sentence production tasks immediately after spoken word–picture matching tasks. This method was based on the assumption that the lexical representation of the target word is activated in advance by performing the spoken word–picture matching, which makes it easier to activate the speech processing. The participants took part in two conditional sessions: the tDCS condition, in which they performed spoken word–picture matching tasks while we delivered the anodal tDCS over the left inferior frontal cortex, and the sham condition, in which we delivered sham stimulation. We hypothesized that the deblocking effect would be enhanced by applying anodal tDCS, compared with the sham stimulation, over the left inferior frontal cortex while patients performed tasks requiring access to semantic representations, so that performance of subsequent naming and sentence production would be improved.

We further applied self-administered tablet-based training, in place of face-to-face treatment by a therapist. Tablet-based language training has recently been reported to be effective for chronic post-stroke aphasia (Des Roches et al., 2014; Kurland et al., 2014, 2018). Fridriksson et al. (2011) showed that combined with tDCS, self-administered computerized anomia treatment reduced processing time during picture naming in participants with fluent aphasia, and its benefits persisted for 3 weeks. Thus, this combination has the potential to become a new rehabilitation therapy for chronic aphasia.

## Materials and Methods

### Participants With Aphasia

We included in the experiment six aphasic participants (five males and one female, aged 50 years–78 years) who had suffered a left hemisphere stroke (Table 1). Our study aimed to facilitate lexical retrieval by language training combined with tDCS; thus, we targeted any aphasia type with impaired lexical retrieval: four participants with Wernicke’s aphasia, one with anomic aphasia, and one with mixed aphasia. We selected participants according to the criteria of being native speakers of Japanese, pre-morbidly right-handed, with normal hearing and normal/corrected-to-normal vision, no psychiatric disease, and with the onset of aphasic symptoms having occurred at least 1 year before. An important additional criterion for applying the deblocking technique was that, while naming and sentence production were mildly or moderately impaired, spoken word comprehension was intact. All participants were strongly right-handed (laterality quotient = +100), as confirmed by the Edinburgh Handedness Inventory (Oldfield, 1971). Their normal hearing had been confirmed by neurological examination using a tuning fork. We conducted the pre-test for word selection at between 2 years, 2 months, and 9 years, 2 months after the onset of aphasic symptoms. We assessed the aphasic disorders immediately before the start of training using the Japanese standard language test of aphasia (SLTA; Brain Function Test Committee of Japan Society for Higher Brain Dysfunction, 2003) by speech-language-hearing therapists. The participants scored as follows (Table 1): 10 points out of 10 (spoken word comprehension—that is, spoken word—picture matching) and 10–17 points out of 20 (picture naming). In comparison, the mean (SD) points in 150 non-aphasic individuals were 10 (0.2) points (spoken word comprehension) and 19.6 (0.8) points (picture naming), according to the manual of SLTA. The Ethics Committees for Human and Animal Research of the National Institute of Information and Communications Technology, Kawasaki Medical School Hospital, Kawasaki University of Medical Welfare, and Okayama Rehabilitation Hospital approved the study in advance. We obtained informed consent to participate in the study from all participants.

**Table 1:** Sociodemographic and clinical data of the aphasic participants.

Participant	Time post-onset	Type of aphasia	Lesion location	Japanese standard language test of aphasia	
				Word comprehension (/10)	Picture naming (/20)
1	9 years 2 months	Wernicke	LSTG, LIFG, and the subcortical	10	16
2	5 years 2 months	Wernicke	LMFG, LIFG, and the subcortical	10	17
3	2 years 2 months	Wernicke	LPT, LRC, WM	10	17
4	8 years 11 months	Wernicke	LSTG, LMTG, LAG, LSMG	10	16
5	8 years 1 months	Anomic	LPT	10	10
6	5 years 9 months	Mixed	LMCA	10	16

*Lesion location: LSTG, left superior temporal gyrus; LIFG; left inferior frontal gyrus; LMTG, left middle temporal gyrus; LPT, left putamen; LRC, left radiate crown; LWM, left white matter of frontal lobe; LMTG, left middle temporal gyrus; LAG, left angular gyrus; LSMG, left supramarginal gyrus; LMCA, left middle cerebral artery. Japanese standard language test of aphasia: mean (SD) points in 150 non-aphasic individuals were reported as follows: 10 (0.2) on word comprehension and 19.6 (0.8) on picture naming*.

### Experimental Design

**Figure 1:**
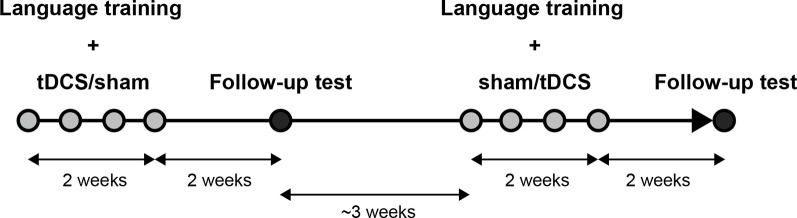
Experimental flow. Each participant took part in two conditional sessions (transcranial direct current stimulation, tDCS and sham). Each session comprised language training with tDCS or sham on four non-consecutive days for 2 weeks and a follow-up test 2 weeks after the end of the training.

We applied a crossover test in the present study, assigning all participants to a tDCS session and a sham session. In the tDCS session, we trained them while delivering tDCS over the left inferior frontal cortex, and in the sham session, we trained them with sham stimulation. Each session comprised language training with tDCS or sham for four non-consecutive days and a follow-up test (Figure 1). We randomized the order of the two sessions across participants. The interval between the last day of training in one session and the first day of the training in another session was about 3 weeks (21–24 days).

### Pre-test for Word Selections

Before the training, speech-language-hearing therapists performed picture naming tests on the participants individually using 466 photographs of familiar objects and collected 80 objects which could not be named correctly for each participant. We selected Japanese words which were object names pronounced with two to eight morae (mora is a phonetic segmental unit of sound of a certain duration; each Japanese syllabogram, kana, basically corresponds to one mora), with high familiarity values (>4.7 on a 7-grade scale) based on the Lexical Properties of Japanese database (Amano and Kondo, 1999), and divided them into the following four sets so that both the number of morae and the word familiarity would be well balanced for each participant: 20 words trained in the DCS session (trained words), 20 trained words for the sham session, 20 words not trained but used in the follow-up test in the tDCS session (untrained words), and 20 untrained words for the sham session. We confirmed the parity in the numbers of morae and the familiarity values among the sets for each participant by the Kruskal–Wallis test (Supplementary Tables S1, S2).

### Tablet-Based Language Training With Deblocking Method

**Figure 2:**
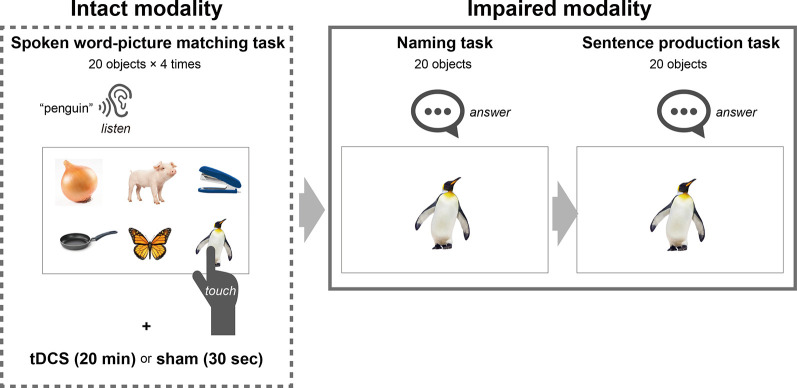
Schematic representation of language training with a deblocking method. The tablet-based training comprised three tasks: spoken word–picture matching for the intact modality, and naming and sentence production for the impaired modalities. In the spoken word-picture matching task, six photos were displayed on each page which contained one object describing one of 20 trained words and five filler objects, and then the trained word was presented by a female narrator’s voice. We instructed the participants to touch one photo corresponding to the spoken word. On each training day, the participants repeated the spoken word-picture matching task for the 20 trained words four times while they were given tDCS or sham stimulation to Broca’s area. After performing the spoken word-picture matching task for 20 min, the participants performed the naming task, which was designed to display one photo corresponding to one of the 20 trained words on each page. We instructed the participants to name the names of the 20 objects orally. After the naming task, they performed the sentence production task which was designed to display one photo corresponding with one of the 20 trained words on each page. We instructed the participants = to produce a simple sentence containing the word corresponding to the presented object. The time limit for each page was 15 s for all three tasks.

We provided an application for a tablet comprising three modules to enable the training of naming and sentence production with the deblocking method: spoken word–picture matching for the intact modality, and naming and sentence production for the impaired modalities (Figure 2). We designed the spoken word–picture matching module to display six photos on each page: one object describing any of 20 trained words, and five filler objects. Three seconds after the presentation of each page, the trained word was presented by a female narrator’s voice. We instructed the participants to touch one photo corresponding to the spoken word within a time limit of 15 s. The next page was presented after the time limit or the touching. On each training day, participants repeated the spoken word-picture matching task for the 20 trained words four times for 20 min while receiving the tDCS or sham stimulation (described below in detail).

We designed the naming module to display one photo corresponding to one of the trained words on each page, which the participant pointed to in the spoken word-picture matching task (Figure 2). For the naming task, we instructed the participants to name the 20 objects orally, each within a time limit of 15 s. We displayed the next page after the time limit or after they touched a button on the screen. We designed the sentence production module to display one photo on each page, as for the naming task. We instructed the participants to produce a simple sentence for each of the 20 photos, which contained the word corresponding to the object, within the time limit of 15 s (for instance, for a photo of an apple, “I ate an apple,” “I like apples,” and so on). In both tasks, we recorded the participants’ responses (i.e., their voices). We displayed the next page either after the time limit or after they touched a button on the screen. We presented the objects representing the trained words in random order for each training day. Thus, the participants repeatedly performed the training of naming and sentence production for the 20 trained words throughout four non-consecutive days (2 days/week) using a tablet (YOGA Tab 3 10, Lenovo).

### Transcranial Direct Current Stimulation (tDCS)

We delivered tDCS by a battery-driven constant DC stimulator (neuro-Conn GmbH, Ilmenau, Germany) through a pair of saline-soaked sponge electrodes (5 cm × 7 cm), with the anode electrode centered over F5 of the extended International 10-10 system for EEG electrode placement, corresponding to Broca’s area, and the cathode electrode over the right orbitofrontal cortex. In the tDCS session, we delivered a 1.5-mA anodal stimulation (0.043 mA/cm^2^) for 20 min while the participants were performing the spoken word-picture matching task with the tablet. In the sham session, the current intensity was the same as that in the tDCS session, but we turned off the stimulator after 30 s without notifying the participant. We started the naming task 20 min after the onset of stimulation in both sessions. While instructing participants on the experiment, they were told that tDCS would be applied for 20 min during the spoken word-picture matching task in the two sessions. Thus, at the time of participation, they did not know that one of the sessions was sham stimulation. After the experiment, we enquired whether they had noticed that they received sham stimulation, but no participant identified if the session was real or sham stimulation.

### Follow-Up Test

In each session, we conducted a follow-up test 2 weeks after the last training to investigate the duration of the effect and the extent of generalization. The participants performed a naming task and a sentence production task for each of the 20 trained words and 20 untrained words. The order of the words was random in each task.

### Data Analysis

We recorded the participants’ answers in each session, and they were judged by one evaluator who was blind concerning the assignment of tDCS and sham sessions. The answers with paraphasia and those over the time limit were judged to be incorrect. For the sentence production task, five speech-language-hearing therapists who were blind concerning the assignment of tDCS and sham sessions judged the answers based on the following two criteria: (1) a correct name and its related word were produced; and (2) a sentence with no semantic and grammatical errors was produced, in addition to (1).

To assess the effectiveness of the training in each session, we compared the accuracy rate at the last training day and the follow-up day, respectively, with the pre-training (i.e., 0%) by a Wilcoxon signed-rank exact test. Furthermore, we assessed differences in the accuracy rates at the first and last training day between tDCS and sham sessions by a two-way repeated measure analysis of variance (ANOVA) with one within-subject factor of the session (tDCS and sham) and one between-subject factor of session order (tDCS after sham and sham after tDCS). We also assessed differences in the accuracy rates on the follow-up day by a three-way ANOVA with two within-subject factors of session and word (trained words and untrained words) and one between-subject factor of session order. Before we applied these parametric tests, we confirmed normality for each data using the Shapiro–Wilk test. We considered a significance level of 0.05 to be statistically significant and performed the statistical analysis with SPSS 24.0 software (IBM Corp.).

## Results

### The Immediate Effect of Training

**Figure 3:**
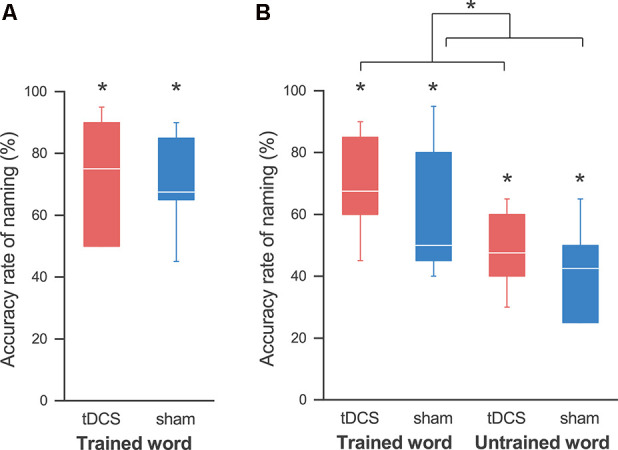
Accuracy rates for the naming task.** (A)** At the last training day, the accuracy rate of naming was significantly higher than that at pre-training in both the tDCS (red) and sham (blue) sessions, and we did not find any effect by tDCS. **(B)** At the follow-up day, we also found an improvement in naming for the trained words in both sessions. Similarly, the accuracy rates for the untrained words were significantly higher than those pre-training in both sessions. A three-way analysis of variance (ANOVA) showed a significant main effect of the session. This reveals that tDCS elicits a greater improvement in naming, regardless of whether the words are trained or untrained. The line in each box represents the median. The top and bottom of each box represent the upper and quartile and lower quartile, respectively. The error bars represent the data range. *p < 0.05.

The naming accuracy of all participants improved after non-consecutive 4-day training in both sessions. With a Wilcoxon signed-rank exact test we revealed the accuracy rate at the last training day to be significantly higher than that in pre-training (i.e., 0%), in the tDCS session (mean ± SE, 72.5 ± 7.5%; *p* = 0.031), and in the sham session (70.0 ± 6.0%; *p* = 0.031; Figure 3A). Two-way ANOVA showed no significant main effect of session (*F*_(1,4)_ = 0.188; *p* = 0.687, partial *η^2^* = 0.045) and session order (*F*_(1,4)_ = 1.492; *p* = 0.289, partial *η^2^* = 0.272), and no significant interaction (*F*_(1,4)_ = 1.688; *p* = 0.264, partial *η^2^* = 0.297). Similarly, two-way ANOVA for the naming accuracy at the first training day showed no significant effect of session (*F*_(1,4)_ = 0.001; *p* = 0.983, partial *η^2^* = 0.0001) and session order (*F*_(1,4)_ = 0.567; *p* = 0.076, partial *η^2^* = 0.586), and no significant interaction (*F*_(1,4)_ = 0.225; *p* = 0.660, partial *η^2^* = 0.053).

**Figure 4:**
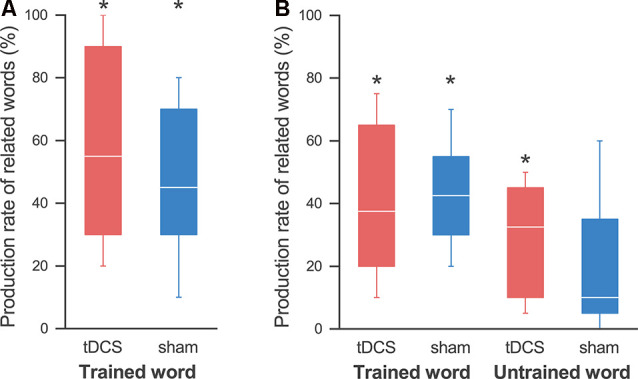
Production rates of related words.** (A)** On the last day, the production rate of the related words was significantly higher than that at pre-training in both the tDCS session (red) and the sham session (blue), and we found no effect by the tDCS. **(B)** At the follow-up day, the production rate of words related to the trained words was significantly higher than that at pre-training in both sessions. On the other hand, we found significant improvement in the production of words related to the untrained words in the tDCS session, but not in the sham session. The line in each box represents the median. The top and bottom of each box represent the upper and lower quartiles, respectively. The error bars represent the data range. *p < 0.05.

**Figure 5:**
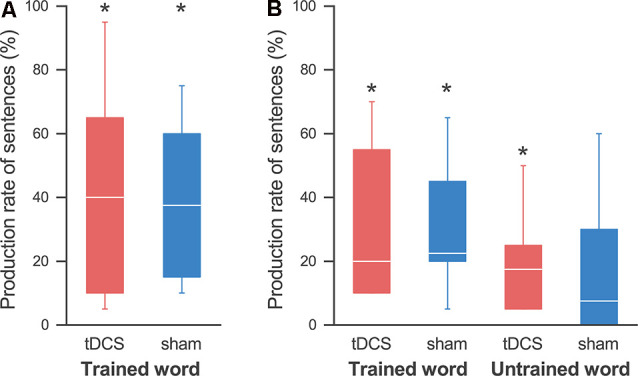
Production rates of sentences.** (A)** On the last day, the production rate of sentences was significantly higher than that at pre-training in both the tDCS session (red) and the sham session (blue), and we found no effect by the tDCS. **(B)** At the follow-up day, the production rate of sentences using the trained words was significantly higher than that at pre-training in both sessions. On the other hand, we found significant improvement in sentence production for the untrained words in the tDCS session, but not in the sham session. The line in each box represents the median. The top and bottom of each box represent the upper and lower quartiles, respectively. The error bars represent the data range. *p < 0.05.

We also detected improvement in the sentence production task after the 4-day training for all participants, although there were individual differences. A Wilcoxon signed-rank exact test showed the production rate of related words in both the tDCS session (58.3 ± 11.8%; *p* = 0.031) and the sham session (46.7 ± 10.2%; *p* = 0.031) to be significantly higher than that in pre-training (Figure 4A). A two-way ANOVA showed no significant main effect of session (*F*_(1,4)_ = 2.481; *p* = 0.190, partial *η^2^* = 0.383) and session order (*F*_(1,4)_ = 0.569; *p* = 0.493, partial *η^2^* = 0.124), and no significant interaction (*F*_(1,4)_ = 0.051; *p* = 0.833, partial *η^2^* = 0.013). Similarly, the production rate of sentences was also significantly higher than that for pre-training in both the tDCS session (42.5 ± 12.8%; *p* = 0.031) and the sham session (39.2 ± 10.8%; *p* = 0.031; Figure 5A). Again, a two-way ANOVA showed no significant main effect of session (*F*_(1,4)_ = 0.180; *p* = 0.693, partial *η^2^* = 0.043) and session order (*F*_(1,4)_ = 0.394; *p* = 0.564, partial *η^2^* = 0.090), and no significant interaction (*F*_(1,4)_ = 0.404; *p* = 0.559, partial *η^2^* = 0.092; Figure 5A).

### The Long-lasting Effect of Training

At the follow-up day 2 weeks after the last training day, the improvement in the naming of the trained words lasted in both sessions: a Wilcoxon signed-rank exact test showed that the accuracy rate was significantly higher than that in pre-training in both the tDCS session (69.2 ± 6.4%; *p* = 0.031) and the sham session (60.0 ± 8.3%; *p* = 0.031; Figure 3B). Similarly, the accuracy rates of naming for the untrained words were also significantly higher than those for pre-training in both the tDCS session (48.3 ± 5.1%; *p* = 0.031) and the sham session (41.7 ± 6.0%; *p* = 0.031), which revealed generalization of the training in both sessions. As expected, a three-way ANOVA showed a significant main effect of the word (*F*_(1,4)_ = 20.840; *p* = 0.010, partial *η^2^* = 0.839), indicating that the accuracy rate for the trained words was higher than that for the untrained words. Importantly, we also detected a significant main effect of the session (*F*_(1,4)_ = 8.205; *p* = 0.046, partial *η^2^* = 0.672), signifying that the accuracy rate was higher in the tDCS session than that in the sham session. There were no significant effects of session order (*F*_(1,4)_ = 0.943; *p* = 0.387, partial *η^2^* = 0.191) or interaction between the three factors.

We also found an improvement lasting for 2 weeks in the sentence production task for the trained words. A Wilcoxon signed-rank exact test showed that the production rate of words related to the trained words was significantly higher than that during pre-training in both the tDCS session (40.8 ± 9.4%; *p* = 0.031) and the sham session (43.3 ± 6.8%; *p* = 0.031; Figure 4B). On the other hand, we found a significant improvement in the production of words related to the untrained words in the tDCS session (29.2 ± 6.8%; *p* = 0.031), but not in the sham session (20.2 ± 8.6%; *p* = 0.063; note that all participants showed some improvements in the tDCS session, while one participant showed no improvement in the sham session). A three-way ANOVA revealed a significant main effect only of word (*F*_(1,4)_ = 13.781; *p* = 0.021, partial *η^2^* = 0.775), but no significant main effect of session (*F*_(1,4)_ = 0.640; *p* = 0.469, partial *η^2^* = 0.138) or interactions.

Similarly, the sentence production rate for the trained words was significantly higher than that during pre-training in both the tDCS session (30.8 ± 9.5%; *p* = 0.031) and the sham session (30.0 ± 8.0%; *p* = 0.031; Figure 5B). We found a significant increase for the non-trained words in the tDCS session (20.0 ± 6.2%; *p* = 0.031), but not in the sham session (17.5 ± 8.8%; *p* = 0.125; note that all participants showed some improvements in the tDCS session, while two participants showed no improvement in the sham session). A three-way ANOVA showed no significant main effect or interaction.

## Discussion

Here we aimed to investigate whether the performance of naming and sentence production in chronic post-stroke aphasias could be enhanced by facilitation training combined with anodal tDCS over the left inferior frontal cortex, conducted on non-consecutive days, compared with non-use of tDCS. Results indicated greater improvement 2 weeks after training with tDCS than that after training with sham stimulation. Regardless of whether the words were trained or not, the accuracy rate of naming was significantly higher in the tDCS condition than that in the sham condition. Also, we found a significant improvement in the production of related words and sentences for the untrained words in the tDCS condition, compared to that in pre-training, while in the sham condition there was no significant difference from pre-training. Our results support the hypothesis that the deblocking effect is enhanced by applying anodal tDCS over the left inferior frontal cortex while patients perform tasks that require access to semantic representations, thereby improving performances for subsequent naming and sentence production, compared with results for the sham stimulation.

On the last day of training, the performance of naming and production of related words and sentences were significantly higher than those in the pre-training in both the tDCS and the sham conditions, and we observed no enhancement in the training effect by concomitant use of tDCS. In the tDCS studies on post-stroke aphasia, picture naming (Monti et al., 2008; Baker et al., 2010; Fiori et al., 2011; Flöel et al., 2011; Kang et al., 2011) and sentence production (Marangolo et al., 2013a,b) have been reported to improve during and immediately after training with tDCS. The most noticeable difference between these studies and the present study was that we did not conduct the training with tDCS on consecutive days. This raises the possibility that tDCS applied on non-consecutive days does not enhance the training effect, but the results of our follow-up test contradict this conclusion because the training with tDCS resulted in a significantly higher improvement than that in the sham condition, at least in the naming task. Another possibility is that the deblocking method might maximally promote individual function, and be unaffected by the tDCS and sham conditions.

We found significant improvement in the naming and generation of related words and sentences for the trained words at 2 weeks after the end of training than that before training in both sessions, and the long-lasting effect on naming was higher in the tDCS session than in the sham session. Our results indicate that tablet-based language training with the facilitation method is effective and that the use of tDCS enhances naming performance. The network of language functions activated by deblocking becomes stable if it is utilized in subsequent training or daily language activities (Tanemura, 1992). The results of the present study suggest that tDCS produces more sustained circuit activation during the deblocking method, resulting in greater improvement on the follow-up day. Because we did not find any significant effect of session order during the training, the enhanced effect by tDCS would last for <3 weeks. Many previous tDCS studies also showed greater improvement in naming after training combined with tDCS (1 week to 1 month after) in chronic aphasic patients (Baker et al., 2010; Flöel et al., 2011; Fridriksson, 2011; Fiori et al., 2013). An important difference from the previous study is that these previous studies conducted the training with tDCS on multiple consecutive days, whereas we conducted training on non-consecutive days. To our knowledge, ours is the first report on the efficacy of tDCS used on non-consecutive days for aphasic training. Our results enhance the viability of language rehabilitation using tDCS, even when consecutive-day training is difficult.

Similar to the results for trained words, we found an improvement in the naming of untrained words at 2 weeks after training in both sessions compared with pre-training, and the tDCS session showed greater improvement than the sham session. This result shows that generalization is produced regardless of tDCS use, but is enhanced by the use of tDCS. Interestingly, we observed generalization in the sentence generation task only in the tDCS session, but not in the sham session. The production of related words and sentences for untrained words showed significant improvement compared with that before training in the tDCS session, but not in the sham session. For language rehabilitation, it is important to enhance the generalization of the training effect. Therefore, we believe that the improvement in the untrained words is a critical result of developing new language rehabilitation using tDCS. Fridriksson et al. (2011) performed computerized anomia treatment (spoken word–picture matching) combined with anodal tDCS over the perilesional brain regions in fluent aphasic participants. They reported a reduction in reaction time during the naming of trained items, compared with sham stimulation, immediately after treatment, and at 3 weeks post-treatment, but no difference for untrained items in both tDCS and sham treatment conditions. The difference in the generalization in their study may be due to the difference of tasks on each training day: the participants in their study performed only the spoken word-picture matching task on each training day, whereas those in the present study performed it as a pre-stimulus for the deblocking method but also performed the naming and sentence production tasks. Marangolo et al. (2013a) showed that aphasic participants produced more content units, verbs, and sentences 1 month after a 10-day conversational therapy treatment during which they discussed the contents of a video clip while anodal tDCS on Broca’s area was applied, and also that the use of tDCS produced improved video clips, which were not used during treatments; thus, tDCS facilitated the generalization of language treatment. From the results of neuroimaging studies, the left frontal cortex is involved in top-down retrieval and selection of contextually appropriate meanings (Thompson-Schill et al., 1999; Badre et al., 2005; Bunge et al., 2005; Gold et al., 2006; Grindrod et al., 2008). Therefore, they considered that anodal tDCS of this area facilitates top-down processing, thereby eliciting the integration of word meanings into an unfolding discourse representation of the context. Based on the previous studies, we considered that in our study, the greater training effect after stimulation of the left frontal cortex was obtained by facilitation of semantic retrieval and/or selection on each training day.

Regarding the limitation of this study, since the number of participants was small, we could not analyze the influence of certain conditions (aphasia type, damaged area, onset period, and so on) on the efficacy of this method. Further study is needed to clarify these points.

## Data Availability Statement

The raw data supporting the conclusions of this article will be made available by the authors, without undue reservation.

## Ethics Statement

The studies involving human participants were reviewed and approved by The Ethics Committees for Human and Animal Research of the National Institute of Information and Communications Technology, Kawasaki Medical School Hospital, Kawasaki University of Medical Welfare, and Okayama Rehabilitation Hospital. The patients/participants provided their written informed consent to participate in this study.

## Author Contributions

AI and JT designed the experiment. AI produced the application for tablets and the material. AM, YI, MT, and KH recruited and selected the participants and performed the pre-tests. AI conducted the tDCS experiments and analyzed the data. The results of the data were discussed by all authors. AI and JT wrote the first draft of the manuscript. All authors contributed to the article and approved the submitted version.

## Conflict of Interest

The authors declare that the research was conducted in the absence of any commercial or financial relationships that could be construed as a potential conflict of interest.
