# Supplementary material for: Enhancement of Facilitation Training for Aphasia by Transcranial Direct Current Stimulation
Source: Front Hum Neurosci. 2020 Sep 11;14:573459. doi: 10.3389/fnhum.2020.573459 (PMC7516201; doi:10.3389/fnhum.2020.573459)
Supplement: Supplementary file 1 [file Table_1.DOCX]

Supplementary Material

**Supplementary Table 1. The numbers of morae of trained and untrained words.**

| Participant | Trained word | | Untrained word | | Kruskal–Wallis test  (*p* value) |
| --- | --- | --- | --- | --- | --- |
|  | sham | tDCS | sham | tDCS |  |
| 1 | 3.9 ± 1.1 | 3.7 ± 1.0 | 4.0 ± 1.4 | 3.5 ± 1.0 | 0.6 |
| 2 | 3.6 ± 0.9 | 3.8 ± 1.0 | 3.6 ± 1.0 | 3.6 ± 1.0 | 0.9 |
| 3 | 3.6 ± 0.8 | 3.7 ± 1.0 | 3.3 ± 1.1 | 3.5 ± 1.1 | 0.7 |
| 4 | 3.6 ± 1.1 | 3.5 ± 0.9 | 3.6 ± 0.9 | 3.4 ± 1.0 | 0.9 |
| 5 | 3.4 ± 0.7 | 3.3 ± 0.9 | 3.5 ± 1.1 | 3.3 ± 1.0 | 0.9 |
| 6 | 3.2 ± 0.9 | 3.2 ± 0.9 | 3.3 ± 0.9 | 3.3 ± 0.9 | 1.0 |

**Supplementary Table 2. The familiarity values of trained and untrained words.**

| Participant | Trained word | | Untrained word | | Kruskal–Wallis test (*p* value) |
| --- | --- | --- | --- | --- | --- |
|  | sham | tDCS | sham | tDCS |  |
| 1 | 6.0 ± 0.4 | 6.0 ± 0.4 | 6.0 ± 0.3 | 5.9 ± 0.3 | 0.7 |
| 2 | 6.0 ± 0.2 | 6.1 ± 0.2 | 6.0 ± 0.4 | 6.0 ± 0.3 | 0.3 |
| 3 | 6.0 ± 0.4 | 6.2 ± 0.2 | 6.0 ± 0.4 | 6.0 ± 0.4 | 0.2 |
| 4 | 6.1 ± 0.3 | 5.9 ± 0.3 | 6.0 ± 0.3 | 6.0 ± 0.4 | 0.7 |
| 5 | 6.0 ± 0.3 | 6.0 ± 0.3 | 6.0 ± 0.4 | 6.0 ± 0.3 | 1.0 |
| 6 | 6.0 ± 0.4 | 6.0 ± 0.2 | 6.1 ± 0.2 | 6.1 ± 0.4 | 0.9 |
